# Essay on Unconscious Cerebration!

**Published:** 1868-08

**Authors:** S. P. Breed

**Affiliations:** Princeton, Ill.


THE
### CHICAGO MEDICAL EXAMINER.

N. S. DAVIS, M.D., Editor.

VOL. IX. AUGUST, 1868. NO. 8.

### Original Contributions.

### ARTICLE XXVII.

### ESSAY ON UNCONSCIOUS CEREBRATION!

Read to the Military Tract Medical Society.

By S. P. BREED, M.D., of Princeton, Ill.

[Published by Request of the Society.]

From the best information we have, the Pythagorians, some four hundred years before Christ, broached the theory that the earth moves.

Aristarchus, of Samos, a Grecian philosopher, is delivered down to us as the principal, *if not the first*, who maintained that the earth turns upon its center, and describes a circle around the sun. Such doctrines exposed Aristarchus to the severest censure of the sectaries of his time. Cleanthes, prominent among his enemies and calumniators, charged him with shaking, with rude impiety, the Throne of Vesta, who was worshipped as the patroness of fixed habitations and civilized life. Her sacred seat was represented to be near the center of the earth, and was declared to be always *fixed*, firm, and immovable. Whereupon, he was accused before the Court of Areopagus, of violating morality, and introducing innovation in religion. Just what punishment was inflicted upon him I have been unable to ascertain, though I have searched diligently through many ancient records, but that it was such as to make his name

*infamous* for *many centuries,* there is abundant evidence. By such unreasonable opposition, and such absurd objections, the the sublime philosophy of Aristarchus (the central theory of celestial science) was suppressed, and effectually held in abeyance *for nearly nineteen hundred* years, until the commencement of the sixteenth century, when Copernicus adopted and improved the hypothesis of the Pythagorians, which made the *sun* the center of the solar system, and the earth to move *not only* around the sun but around its own axis also; thus establishing the system of astronomy, which still goes by his name, and is, now, universally received as the true theory.

Apprehensions, arising from the novelty of his opinions, and also, doubtless, from the fate of Aristarchus, had, it is said, almost brought him to drop all thoughts of publishing his work, which had *lain* in his escritoir, not nine years *only*, which is the time Horace prescribes, but *nearly four times nine years.*

At length, however, its contents becoming known, and its merits canvassed and appreciated by a few of the more learned and intelligent, through the importunity of these, and his personal friends, he was, at last, prevailed upon to let it come out. But, *when* a copy of it was presented to him, *so great* was his *agitation*, in view of the *odium certain* to be cast upon him, and the relentless persecution with which he would be pursued, that he was *presently* seized with a violent effusion of blood, which put an end to his life on the 24th day of May, 1543.

Gallileo, nearly a century after, in 1612, observed some spots upon the *sun*, and, in his printed discovery of them, he ventured to reässert the truth of the Aristarchan or Copernican theory; and he brought forward several new arguments to confirm it. This startled the Jesuits, who, thereupon, procured a citation for him to appear before the *Holy Office* at *Rome*, in 1615, where he was charged with *heresy* for maintaining these two propositions, *viz.:* 1. That the sun is the center of the world (universe), and immovable by a local motion; and, 2. That the earth is not the center of the world (universe), nor immovable, but does actually move by a diurnal motion. The first of these propositions was declared to be *absurd*, false in philosophy, and formally heretical, being contrary to the *express Word of God.* The second was also alleged to be philosophically false, and in a theological point of view, at least, erroneous in faith. "The Inquisition" pronounced sentence against him and his book. They obliged him to abjure his erroneous views, in the most solemn manner, committed him to the prison of their office during pleasure, which was until 1634, *nearly twenty years*, and his books were *gathered* and *burned* at *Rome*, and the *ashes scattered to the four winds of heaven.*

Notwithstanding this solemn abjuration, and the vigorous efforts of the *priesthood* to suppress his views, and keep the people in ignorance of them, the *arguments*, upon which his hypothesis *stood pillared*, became known, and those who were capable of judging soon saw that the evidences of their truth were such as to *command belief.* They saw that this theory explained many of the phenomena of the heavenly bodies, such as the seasons, eclipses, etc., which were quite inexplicable by the old Ptolemaic system, and that they addressed themselves so directly to the reason and judgment that they would be, *must be*, believed. Seeing and believing thus, they were encouraged to discuss these propositions among themselves, cautiously and prudently at first, but finally openly and boldly, till at length they were gradually accepted by the learned and more intelligent, and, finally, adopted as the basis upon which has since been erected the *solid and noble superstructure of modern astronomy.*

When the superstitious world found that they were no longer able to gainsay or withstand the arguments advanced in support of these doctrines, and also foresaw in their general adoption, while they persisted in their hostile attitude, the certain destruction of their cherished systems of false religion, they immediately changed their tactics, dropped their hostile guise, and rapidly reconstructed their religious views, so as to make them harmonize with these stubborn facts.

In a review of the history of science, in its progress up to its present status, one is struck with no little wonder, in tracing, step by step, the advance of truth through the devious mazes of error, doubt, and prejudice, at the character of the opposition it has often met, and the great length of time this advance has been arrested and kept at a stand-still by the blind opposition and bigotry of those who should have been the first to herald its approach, and most eager to push it forward.

It is, moreover, a matter of no little surprise to us, *now*, as we look back from our present stand-point over the past, to see how very nearly some of the early philosophers came to comprehending many of the fundamental principles of the various sciences, and we are animated with a feeling of *holy* indignation to observe how long these truths were obscured and held in check by suspicious and designing men. It strikes one with wonder, also, now, to see the singular obtuseness of the masses oftentimes in apprehending the facts and arguments which it seems to us, should have been sufficient to arouse their minds to sensibility and investigation. But so it is, the progress of scientific truth was so often retarded by the sinister opposition of the suspicious and the absurd objections of the ignorant, that much valuable time, great patience, and extraordinary energy were required to overcome the obstacles constantly thrown in its path; and it was not until the great body of the people became so enlightened as to decide these questions for themselves, instead of looking to *priests* and *rulers*, that science has made any considerable progress among them.

Wm. Godfrey Leibnitz, baron of Leipsic, as long ago as 1685, in his Protogea, advanced the Igneous theory of geology, by which he explained the formation of the earth, and the subsequent changes of its crust, corresponding almost exactly with the one so generally adopted *now* by geologists under the name of Igneous Agency. This theory, so early enunciated, must have been still-born, for we hear nothing more of it until 1749, nearly a hundred years afterwards, when Buffon, the French naturalist, produced an elegantly written hypothesis upon the formation of the earth, based chiefly upon the views of Leibnitz. These views, thus exhumed, gave great offence to the Faculty of Theology at Paris, and he was obliged, like Gallileo, to recant opinions which are now maintained by all geologists. So the matter rested for almost another century. In the meantime "the world moved on." Investigations by scientific observers, in different parts of the world, were carefully made; facts bearing upon the subject were accumulated in great numbers, until, by careful comparison, free and thorough discussion, sufficient data were procured to enable the truth of the Igneous theory to be made out.

But the pseudo-religionists, *now* as in the former case, when they found that they were no longer able to meet the arguments in support of geology, nor make any effectual stand against the progress of these scientific truths, and, moreover, when they saw the ground upon which they stood fast giving away beneath their feet, and their whole structure of false philosophy *tottering, then*, very reluctantly, made a virtue of necessity, and did as their predecessors had done before, in regard to the Copernican system of astronomy, ceased their blind opposition, and again reconstructed their religious theories in accordance with the inexorable logic of events, and made them harmonize with those inflexible facts they could no longer successfully controvert.

The learned mathematician Leibnitz, in 1685, also advanced the startling doctrine of "Unconscious Cerebration." This announcement was, it seems, still more unfortunate than his new doctrine of geology. Nothing was said, and very little known, about it after its declaration, until some twenty years ago, when Dr. Carpenter, without knowing that he had been preceded, advanced the theory of Unconscious Cerebration to account for some anomalies of mental action. After satisfying himself in regard to the probable truth of the theory, he submitted his views to two of the profoundest thinkers of the age, Sir Wm. Hamilton and Mr. John Stuart Mill. From the former he learned that the same doctrine had been advanced some two hundred years before, by Leibnitz. By the latter, he was assured that the unconscious development of a subject of thought was so familiar to him, that, when he found it difficult to pursue an inquiry further, not seeing his way clearly through its entanglements, he was accustomed to lay it aside for weeks, or even months, and to devote himself to other objects, with the full expectation of being able to pursue his first investigations with diminished difficulty whenever he resumed it. Notwithstanding this high authority, this doctrine has made very little progress in the minds of the learned psychologists of the day. Perhaps *the great reason* why this doctrine has not been accepted by the scientific world arises from the indisposition to regard *mind* in connection with *organization*, *from the fear* of favoring the *opinion* that *mind results therefrom.*

Moreover, many are probably deterred from investigating this subject as they might, from the jealous fear always actuating the opposers of science, of finding something that will disturb the established dogmas of the day. But as honest searchers after truth, we should not shun a careful investigation of so important a matter, nor should we allow our previous opinions or prejudices to forestall our judgment, or divert the current of our thoughts from tending whithersoever the evidence may carry them. If we do this, I apprehend we shall find that *mind* which, at a first view, at times, appears so absent and so abnormal is, in reality, *after all*, no exception to all other phenomena in nature. The more we study this subject unbiasedly, I think, the more certainly will we be led to the conclusion that the mind is as much under the presidency of law as any other function of the body. Every thought, every impulse, and every proclivity is but the legitimate result of a definite cause, and these causes follow each other, and their antecedent causes, with as much regularity, precision, and certainty, as any such necessity in the physical world. There are subtle laws pervading every department of mental activity—running through and through all the avenues of the mind, conscience, reason, judgment, and the affections which control, direct, regulate, and moderate or intensify all our schemes, speculations, and determinations. From these all-permeating, yet unseen, laws, result all our confidence in the virtue and fidelity of our fellow men. From these latent forces arise all the exalted aims, the noble and sublime achievements of the great and the good as well as the low and the bad. Upon them rest all our attachments, affinities, and cherished relations of mind with mind. The mental *eye* looks out into the world, around, through these variously colored media, as a kind of kaleidoscope which may, by reflection, refraction, transposition, or polarization, magnify or diminish the objects and views, giving light and shade, tint and color, with as much respondency to law, with as much certainty and regularity as any phenomena of light and optics in natural philosophy.

As the natural eye, in looking through an uneven pane of glass, will see objects distorted and comic, so will impressions upon the *cerebrum* so modify its nutrition, by some delicate and peculiar molecular change therein, as to change the whole mental character, by *intensifying a certain class of impressions* upon the consciousness of the individual, to the *exclusion of others.* Hence, we have mental as well as optical illusions; objects party-colored and distorted, magnified or diminished, so as to control opinions, produce unnatural attachments, sharpen censures, or infuse unwonted severity into penalties and judgments.

The cerebrum in man, therefore, is a wonderful instrument of capabilities, caprices, and powers. It is owing to the much larger development of this part of the brain over the lower animals that gives to *man* his preëminence over all other orders and species of the animal kingdom. By reference to comparative anatomy, it will be found that the relative *proportion of this organ* diminishes as we descend from the higher mammalia to the lower, from them to the birds, from thence to the reptiles, and from these again to the fishes.

The brain is placed in relation with the outside world through the senses. The various sensory impressions are thence conveyed, through their respective and appropriate nervous filaments, to the sensorium, located in the sensory ganglia. Under the term sensory ganglia, may be included that assemblage of ganglionic masses lying along the base of skull in man, and partly included in the medulla oblongata, in which the special senses, seeing, hearing, feeling, tasting, and smelling, have their central terminations.

It seems to be a peculiar arrangement of the nervous apparatus, that excitor impressions should travel upwards, if they meet with no interruption, until they arrive at the cerebrum. When they reach the sensorium, they make an impression on the *consciousness* of the individual, and thus give rise to a sensation; and the molecular change thus induced, being further propagated from the sensory ganglia to the cerebrum by reflex action, becomes the occasion of the formation of an *idea.* If, with this idea, any pleasurable or painful feeling is associated, it is *called* an *emotion*, and, either as a simple or emotional idea, it becomes the subject of intellectual operations, whose final issue may be a volitional determination which may be exerted in checking or producing muscular motion, or in controling or directing, to some extent, the current of thought.

On the reception of sensory impressions the changes which they produce in the sensory ganglia give rise to a new excitement of nerve force, which is propagated along the *ascending fibers* to the *vesicular matter* that forms the *surface* of the cerebral hemisphere; and *it is not* till they *arrive* at the *ulterior termination* of these *fibers* that their *impressions produce* those *changes* which are *instrumental* in the *formation* of IDEAS, and subsequently in the *higher* and *more complicated intellectual operations.* These intellectual operations, *themselves now*, become the source of new changes in the condition of the vesicular matter of the nervous substance, and an excitation of nerve force takes place *here* as their result, which, transmitted downwards to the sensorium, gives rise, through it, to appropriate respondent movements. The *sensory ganglia*, therefore, let it be remarked, constitute the *seat* of *all consciousness*, *not only* for *impressions* on the *organs* of *sense*, but *also* for *changes* in the *cortical substance* of the *cerebrum.* So that, it is not until these changes have reacted downwards upon the sensorium that we have any knowledge or consciousness of the formation of ideas, or of any intellectual processes which may be going on there. The cerebrum, therefore, on the receipt of impressions, reflected from the sensorium, immediately commences the operation of working up the materials submitted to it. This process may continue unconsciously to us, unless interrupted by new impressions, in which case the former process seems to be temporarily arrested, until the import of the new intelligence is fully made out, when the *cerebration* is resumed, with such modifications as the new information may seem to require, or pursued after the tenor of the former train. Hence, we often see a *trivial incident*, seen perhaps a thousand times before, without making any notable change in the current of thought, produce the most striking, unlooked for, and important results. An intercurrent thought thus striking the mind at a particular juncture, incidentally, while it is in a peculiarly excited state of inquiry, when the cerebrum seems to be casting about for more facts, and the thoughts break loose, as it were, from former combinations, reaching and *feeling out around* for more materials; *it is at such* a *time* that *some mere trifle*, *some apparently insignificant circumstance*, will wholly *change* the *current* of *thought*, and *divert it into* a *fruitful field* of *important discovery*.

Sir Isaac Newton had, doubtless, seen apples fall a hundred times before, but as his mind was not in this "*statu-nascente*" condition, no important results followed. But *now*, when his mind was on the *qui vive* for new truths, new facts, to bring to the test certain theories evolved from previously received data, *then it was* that this little incident, the most trivial in nature, coming in just at the right time, and springing a felicitous train of thought, gave such a new direction to his investigations as, followed up to their legitimate conclusions, resulted in the *most stupendous discovery* of *any age*. Newton made this discovery, not because he *willed* it, but because he could *not help it*. It was, in a very important sense, an unavoidable result, the evitable evolution of his philosophic brain. The office of the organs of sense *is simply* to supply the crude materials, while it is the province of the cerebrum to receive and work up those materials, solve the problems, and transmit the results down to the sensorium, from whence they are utilized in the practical voluntary affairs of life.

The cerebrum may not inaptly be compared to a jury, while the organs of *sense* may represent the *witnesses.* The jury, after hearing the evidence, retire to a private room, where, by themselves alone, they compare notes, weigh the evidence, and finally work up the case into a verdict. So it is with the cerebrum. She takes particular *note* of all impressions transmitted to her from the organs of sense, and having thus supplied herself with all the evidence she can obtain, she modestly retires to the secret chamber of unconsciousness, where, by a thorough process of careful comparison, sifting, weighing, and discrimination, a *decision* is *reached*, or, in case of no decision, which sometimes happens, then, in this event, either the judgment is suspended for further testimony, or the *jury hangs*—when the matter is indefinitely postponed. It may be remarked, that, with the great majority of mankind, on some of the most important questions of practical utility and philosophic interest, either there has been a premature decision, or the *jury has hung.*

There is much ground for the *belief* that every sensory impression which has once been recognized by the perception, is registered (as it were) in the *cerebrum*, and may be reproduced at some subsequent time, although there may be no consciousness of its existence in the mind during a long intermediate period. Instances are of frequent occurrence in which ideas come up before the mind during delirium or dreaming, and are expressed at the time, although the individual may not be able to remember ever to have heard them before, they yet having been proved to have been heard at some long antecedent period. Dr. Carpenter relates the case of a lady during a delirium of fever who continually repeated sentences of Hebrew and Chaldaic, of which she stated herself, on recovery, to be perfectly ignorant; but on tracing her former history it was ascertained that, in early life, she had lived, as servant maid, with a clergyman who used to walk up and down the hall of his dwelling repeating aloud these passages, which she must have retained in her memory unconsciously to herself.

While preparing this essay I was requested by Dr. Anthony to visit his father-in-law, who was then lying very sick. This gentleman was between seventy and eighty years old. In relating the history of the case, the Doctor said that his mind had been failing for several months. Among the first signs of mental aberration they noticed that he talked about, and manifested great anxiety in regard to, the *cattle* and *sheep* on the farm (by which I knew at once that he had been a farmer), which, he said, were not properly cared for. He had not lived on a farm, nor had anything to do with one for many years. He often inquired about his children, how they were to be supported and educated, though they were all quite grown up, and well settled in life. It is noteworthy that these were the very subjects that occupied his mind some thirty years before. He imagined, also, that he had to take the care of, and support, a colored woman and three small children, which gave him great concern. He talked much about their destitute condition, and repeatedly urged that they should be immediately attended to. When I inquired whether he had ever in his former life had destitute colored neighbors appealing to him for assistance, I was answered, "No, but that he used to be an ardent Abolitionist, and always manifested great sympathy for the colored race in a state of slavery." Here, then, you see we have another striking instance of a long-registered impression spontaneously reproduced, after the lapse of some thirty years, during the general wreck of the mental faculties.

Of the precise nature of the changes by which sensory impressions are thus preserved, we are, in the present state of psychological science, entirely ignorant, but it must be somehow intimately connected and interwoven with the nutrition of the cerebrum, since we do know that alterations in that structure have a marked effect upon the memory.

It is no very uncommon thing for a person to dream the *same thing* over the *second time*, or in a *second dream* to *renew* a former train of thought, taking up the thread just where it was left off in a prior dream. Indeed, some of the most complicated problems in mathematics have been solved during sleep, which had puzzled the brain for hours previously. The process of solution of a difficult problem, interrupted, unfinished, or left because the person could not see his way through it clearly, has been resumed and completed during sleep, by the student rising at night, in a state of somnambulism, and committing it to writing, and the person again retiring to bed. In the morning the student was greatly surprised to see the problem wrought out, *in extenso*, in his own handwriting. So well understood is this matter of unconscious cerebral activity that many persons, when they have a tedious and difficult question to solve, which they cannot readily do, they choose rather to leave it to be wrought out in this way, feeling confident, from past experience, that it will be done. Even the aborigines of our country seem to have some knowledge of this anomaly of mental action, for it is said that they make it a point never to decide a weighty and important matter until they have deliberated well and slept upon it.

It is, in fact, the province of the cerebrum *to think*, as much as it is the office of the lungs to decarbonize and oxygenize the blood, or the stomach to digest food, or the liver to secrete bile. These functions are all carried on in much the same way, under one uniform law of cell-action. Neither one of these functions is entirely under the control of the *will*. All are more or less involuntary. The healthy brain, supplied with suitable materials and conditions, *will think*—think automatically and continually, whether we *will* it or not. We may, it is true, by an arbitrary exercise of the will, modify and control the *current* of our *thoughts*, and educate the mind to certain methods and modes of thinking, until, by long discipline in the habit of close, careful, consecutive thinking, we may acquire great power of reason, and may thus accomplish astonishing results, but *after all this* is *admitted, it is* only when we *intentionally* divert the current of our thoughts from the *channel* in *which they were running*, when we determine to put our mind in operation in some *particular* way or manner different from what it is acting, that we can be said to use the *will* in the act of reasoning; and this arbitrary exercise of it I believe to be much more rare than is generally supposed. Hence, then, we seem justified in *affirming* that the cerebrum may, and very often does, act upon impressions transmitted to it, and may and does actually elaborate results, such as might have been attained by a volitional direction of our minds to the same subjects without any *consciousness* on our part. The experience of most men will readily furnish numerous instances of this kind of cerebral activity.

As the *spinal axis never sleeps*, but, by its constant diffusion of nervous energy, keeps up throughout the body that persistent, tireless state of muscular *tension*, so necessary to the support of the framework and the internal viscera, so do we believe that the cerebrum is constantly acting its part, continually evolving thought so long as the vital forces, the vaso-motor and cell-action continue. If the circumstances connected with the thoughts are such as to favor their reaction downwards upon the sensorium, we are *then* made conscious of them, otherwise we are not. When the avenues, the organs of sense, are closed, or the susceptibility of the sensorium to external impressions is temporarily, and more or less completely, suspended, as in profound sleep, or when its functions are held in abeyance, by the preöccupation and concentration of the mind upon one subject in which its whole faculties are engrossed, the cerebrum *then also* acts unconsciously to us. The *mode* and *character* of the *thoughts* are, therefore, in a great measure, the consequence of the reaction of the cerebrum upon the circumstances which call it into play. While the cerebral, like all other functions of the body, is subject to periods of greater activity and partial repose, it by no means follows that there is at any time a total cessation from all activity in the case of the cerebrum any more than the spinal axis.

Intellectual labor is always, sooner or later, attended with a sense of fatigue, and is also accompanied with cell-metamorphosis, common to all other structures and physical exercises. The products of the disintegration of the brain are always found in the excretions of the body after severe mental labor.

In the delirious ravings of intoxication and of fever, or the perverted reason of the lunatic, we have the same evidence of cell-change that we do in the sayings and doings of the same individuals in a state of health, or normal condition; and I have often thought there is a striking analogy between tissue formation and ideation.

The primary fundamental principle of all organized structures is cellulation. In the normal state these cells are of a determinate character, and a particular kind. The cells are regular and uniform, and the process of cellulation proceeds after a particular type, and goes through the necessary changes successively until they arrive at their ulterior destiny of healthy structure. But in disease, as in cancer, for example, the cells are quite irregular, caudate, fusiform, seniform, spindle-shaped, etc., while they possess increased formative activity. There seems to be an effort to produce healthy tissue, but the action is modified by occult intercurrent causes so that no healthy structure is formed. So it is in delirium and insanity, we have cerebration disordered, visions distorted, conceptions abnormal, sights unusual, figures fantastic, but it is evident that there is a disposition all the time to ideation more or less correct and coherent.

Prof. G. B. Wood, of Philadelphia, once attended a portrait painter through a period of delirium tremens, who, on recovery, in compliance with the Doctor's request, executed a beautiful painting, in which he exhibited, on canvas, the figures and images so indelibly impressed on his memory during his illness. There was one striking peculiarity of this painting. It was this: While part of almost every figure was natural and correct, other parts were as invariably distorted, unnatural, and fantastic; as, a man with the head of a horse, a dog with one side of his head swelled out into irregular and huge proportions, and altogether abnormal; or some *fantastic appendage* to a *natural figure.* Dr. Wood, when he came to his lecture on Delirium Tremens in his course, used always to *present this picture to his class*, and I was told that he prized that picture more than any other in his *museum.* To the psychologist it was, indeed, a very interesting painting. So it is, then, to use a strong synecdoche, a *thought* is but the *bursting of a cerebral cell!* As muscular contraction is effected by the rapid rupturing and reformation of sarcous cells by *nerve force;* so is ideation and ratiocination but the result of the moulting and regeneration of cerebral cells, under the influence of nerve force; or, as the muscles are the instruments for the correlation of nerve force into mechanical power, so is the cerebrum the substratum or instrument through which nerve force is converted into mind. If the brain be healthy and the blood pure, and the circumstances surrounding, and calling it into action, normal, the mental imagery will be perfect, and the *idea* will come forth *full*, *plump*, and *round*, like a *precious coin struck* from the *mint*, and will pass current in the world of thought. But if the brain be diseased, or the mental stimulus, the blood, be loaded with noxious and poisonous matters, *then*, the cerebration will surely be abnormal.

If it be objected that we cannot understand just how nerve force is converted into mind, we might reply, that, neither do we yet quite understand just how *any one* of the physical forces is metamorphosed into *another*. The *modus operandi* of the conversion of electricity into magnetism, and nerve force into mechanical power, is just as mysterious and incomprehensible as the merging of nerve force into mind. So it is, also, with the other forms in which force manifests itself. We are able, however, to discover the apparent annihilation of the *one*, and, by careful observation, we have been able to estimate beforehand the absolute equivalent of the other.

When we have learned more about the dynamics of the nervous system we shall be better able to understand and explain what now appears obscure. If the objector will please to explain the *modus operandi* of the conversion of *nerve force* in the *inferior animals* into INSTINCT or *brute mind*, then, I will, by the same parity of reasoning, explain the *correllation* of *nerve force* into *mind force* in MAN. The *instinct-mind* and *sagacity* of the *brute differ* from the *mental faculties* in *man* in NO OTHER *respect* than that of *degree*. The cerebral organs adapted to *similar functions* are singularly *analogous* in *them all.* When we fully understand the *one*, we *shall have* a *sure* key to the explanation of the other. It is as unnecessary to call in the aid of a "*Deus ex machina*" to help to explain the phenomenon in the *one case* as the *other*. The forces of mind, and the elements of matter, are, in both cases, taken from the *universe with life*, and *restored* to it at *death*. Life and death are antithetic, and *exactly opposite terms*. Life kindles our faculties into conscious existence, and death destroys what life enkindles, so that death must place us back "*statu quo ante vivum*." As we can trace our identity no further back than the commencement of organic life, so does it follow, logically, inevitably, that we will not be able to trace it forward into the future further than death. The idea that man may retain the power of thought after the destruction of the brain, is too absurd to bear the test of careful scrutiny. It is unphilosophical, and contrary to all analogy. It is a figment of the imagination, and a relic of barbarism and superstition.

It is an interesting fact, and one that has much bearing on involuntary cerebration, that some of the most celebrated men for intellectual and artistic ability, have been *mere imbeciles* on all other subjects except those in which they thus excelled. Dr. Carpenter gives two cases, in which the mental action which evolved the result, seems to have been of an automatic character. All accounts of Coleridge's habits of thought, as manifested in his conversation (which was a sort of thinking aloud), agree in showing that his train of mental operation, once started, went on of itself, sometimes in the original direction for a long distance, sometimes with a divergence into some other track, according to the suggestions of others, or circumstances. His whole course of life was one continued proof of the weakness of his will; for, with numerous gigantic projects continually in his mind, he could never bring himself seriously to attempt any one of them; and his utter deficiency in self-control rendered it necessary for his welfare that he should yield himself to the control of others.

The composition of the poetic fragment "Kubla Khan" in his sleep, is a typical example of automatic mental action; and almost his whole life might be regarded as a sort of waking dream, in regard to the deficiency of that self-determining power which is the preëminent characteristic of every really great mind.

The whole artistic life of Mozart, from his infancy to his death, may be cited as an example of that spontaneous or automatic development of musical ideas, which expressed themselves in the language appropriate to them. When only four years old he began to write music, which was found to be in strict accordance with the rules of composition, although he had never received any instruction in them. And when engaged, in adult life, in the production of those works which have rendered his name immortal, it was enough for him once to fix his thoughts in the first instance upon the subject (the libretto of an opera, for example, or the words of a religious service), so as to give them the requisite start or direction, and *then* they flowed onwards without any effort of his own, so that the whole of a symphony, or an overture, would be developed in his mind, its separate instrumental parts taking their respective shapes without any intentional elaboration. In fact, the only exercise of the will that seemed to be required on his part, consisted in noting down the composition when complete. It is recorded of him, that, being once asked by an inferior musician how he set to work to compose a symphony, he replied, "If you once think how you are to do it, you will never write anything worth hearing. I write because I cannot help it." Mozart, like Coleridge, was a man of extremely weak *will.* He could neither keep firm to a resolution, nor resist temptation, and, when not under the guidance of his excellent wife, was the sport of almost every kind of *impulse.* But there probably never was a more remarkable example than his musical career presents, of the automatic operation of that creative power which specially constitutes genius, and his life is altogether a most interesting study to the psychologist.

But there is an instance of a somewhat similar kind, later, and in our own country. You have all doubtless heard of "Blind Tom," the negro boy, who is an *idiot* on all subjects but music. He, however, possesses extraordinary powers of mind on this *one* subject. He can execute the most difficult and complicated pieces of music after hearing them *but once.* It is claimed that he does this from memory, and his inherent and intuitive sense of the beautiful and appropriate in music. I am indebted for the following facts to Mr. G. Gilbert Gibbons, Esq., of Princeton, who, in his able argument in defence of John Card, being tried for the murder of his wife, stated, that, at one time, while in Chicago, he attended a public exhibition given principally for the purpose of testing the musical powers of the negro boy "Blind Tom." Baumbach, the pianist and composer, played, for the first time, one of his most difficult and lengthy pieces. "Blind Tom," on being brought into the room. as soon as the music began, commenced and continued whirling rapidly around on one foot, until the music ceased, when, to the astonishment of all present, he seated himself at the piano, and, not only played the piece through without making any mistakes, but according to Baumbach's own admission, and the universal opinion of the audience after the performance, he actually improved upon the author himself, in purity of tone, accuracy, delicacy, and celerity of touch, and felicity of expression. Many such examples, doubtless, might be given to show that the cerebrum has a way of its own of doing business independent of the will.

The molecular changes constantly going on in the cerebrum as inevitably produce thought as the same molecular changes in the spinal axis produce nerve force, or the cell-action in the liver produces bile, or the same action in the glands produces the various secretions of the body. The difference in the result is owing *alone* to the difference in structure and the circumstances. In each case there is a special structure adapted to the performance of a special function. Nerve force is correlative mind force, vital force, electric force, etc. In fact, it may be declared that there is *but one force* in nature, and that force is *always acting* in *some way* or *other;* and, moreover, is *not subject* to *either increment* or *diminution.* Matter and force, therefore, are eternal and forever the same in amount. One is as equally indestructible as the other. It follows, therefore, that, when one form of this ever-changing force is metamorphosed into some other equivalent force, and, hence, seems to be annihilated, it is in reality *only acting* in some other way—appearing in some other form. Force can neither originate, *de novo*, nor cease to operate in some of its protean forms. Thus, when motion is retarded by friction, *heat* is generated. If the rubbing surfaces are heterogeneous, electricity, as well as heat, is the result.

The fact that one force can, *at will*, be transformed into another, and the equivalent of the new force be determined beforehand with mathematical certainty, marks an era in Physics, Physiology, and Psychology.

Prof. Samuel Jackson's discovery of the correlation of forces, and Schwann's discovery of the animal (I might say the universal organic) cell, are destined to effect as great a revolution in physiology and psychology as the theory of Aristarchus and Copernicus did in astronomy, or that of Leibnitz and Hutton did in geology. "Verily, the world moves."

Planting our feet firmly upon these "look-out mountains" of physical science, as we survey, from this new vantage-ground, the more airy regions of psychology, we may smile at the impotent assaults of, and bid defiance to, our *old enemies;* while they, perhaps, recovering from their temporary dismay and discomfiture, rallying once more in defence of their long-cherished dogmas, marshalling again their scattered hosts for the final *onset*, we expect to see thousands of untried steel leap forth from rusty scabbards in the defence of *truth*, and, by the collision of opposing weapons, *one resplendent blaze* of *intellectual light* and *moral grandeur* shall be *enkindled*, waxing brighter and brighter, until every species of vice and error shall be dispersed by the *irradiating* beams of truth and reason. Then shall the disciples of a *vague* and illusive religion, the victims of error and superstition, and the votaries of Transcendentalism and Empiricism, catching a glimpse of their own *mental obliquity*, startled by a *view* of their own *perverseness*, rush with horror from the ghastly monsters they have been so long straining to their bosoms; *recoiling*, catch the inspiration of the age, vitalized and electrified into a new existence, still again readjust and reconstruct their falling systems and visionary theories, so as to make them *harmonize* with the inexorable logic of these scientific discoveries and a rational philosophy, and be constrained to acknowledge that "*the world moves.*"

